# Editorial: Varieties of agency: exploring new avenues

**DOI:** 10.3389/fpsyg.2025.1572561

**Published:** 2025-03-04

**Authors:** Nivedita Gangopadhyay, Franz Knappik, Vivian Puusepp

**Affiliations:** ^1^University Library, University of Bergen, Bergen, Norway; ^2^Department of Philosophy, University of Bergen, Bergen, Norway; ^3^Department of Philosophy, University of Tartu, Tartu, Estonia

**Keywords:** agency, 4E cognition, digital technologies, artificial intelligence, affordances, perception, embodiment, non-human agency

The Research Topic “*Varieties of agency: exploring new avenues*” is inspired by contemporary theoretical and technological developments in interdisciplinary research on agency, spanning philosophy, psychology, neuroscience, information technology, artificial intelligence and social anthropology. Much of this research brings us back to age-old conceptual questions such as—what is agency? Our starting point in embarking on this Research Topic is to situate the conceptual question of “what is agency” within two intersecting contexts, namely,—(i) the debates in interdisciplinary philosophy of mind and cognitive science between 4E cognition (i.e. the view that cognition is embodied, enacted, embedded, and extended) and its critiques, and (ii) the rapid development and proliferation of digital technology and artificial intelligence that now pervade almost all aspects of our daily lives, raising fundamental questions about who we are—both at an individual level and at a societal level.

The contributions in the Research Topic represent diverse approaches ranging from contemporary cognitive science and philosophy of mind over psychology, anthropology and neuroscience to information technology and artificial intelligence. While some articles directly address the issue of agency, others choose specific themes that are tightly coupled to discussions of agency in a 4E framework—such as perception and embodiment.

Among the papers that directly focus on agency, Wong addresses the timely question of whether AI systems are agents. Rather than arguing for a particular answer, Wong aims to clarify the question and the conceptual resources we have for dealing with it. To this end, he distinguishes between architectural and behavioral approaches to the demarcation of agency—where the former locate the hallmark of agency in forms of organization characteristic of paradigmatic agents, viz., adult humans, while the latter use behavioral criteria like the indistinguishability from instances of bona fide agency (as in the Turing test), or the applicability of the “intentional stance” (Dennett). Wong argues that to decide whether AI systems are agents, both approaches must be combined, and warns against chauvinistic assumptions that prejudge the question against AI agency.

The anthropocentric bias that Wong points to here has been a longstanding target of critique in ecofeminist thought. In her contribution, Trächtler draws on the work of Donna Haraway to challenge widely held basic assumptions about agency, in particular, the tendency to understand the contexts in which human agents find themselves as a mere passive background to their actions. In contrast to this conception, Trächtler explores the theme of the world as a non-human agent, adopting a critical stance on so-called “objective” knowledge of the world, for example as upheld in the natural sciences. The article presents a nuanced discussion of rethinking scientific objectivity about the world, examining how far one can actually consider the world as an agent in terms of epistemically and politically effective agency.

A number of contributions also revisit the theme of “affordances” that has been a corner-stone in 4E cognition as a concept that establishes an intrinsic connection between the agent and the world. Revisiting varieties of agency leads several authors to revisit the concept of affordances—a concept that connects the agent and the world. Thus, Stankozi takes up the discussions of how the agent chooses between competing affordances and proposes that affordance competition drives the process of imagination, thereby linking discussions of imagination to those of affordances. Hansen proposes a strong perceptual account of affordances to explain the perception of visually indistinguishable objects that belong to categorically distinct high-level kinds. Hansen's account strengthens the notion of affordance perception by applying it to long-standing debates about objects that appear visually indiscernible but differ in their underlying nature, such as a real lemon and a lemon-shaped soap bar.

Several contributions directly address core topics from discussions of agency in the framework of 4E cognition, such as situatedness and embodiment. Heijmeskamp explores the issue of how agents react to affordances by focusing on a conceptual discussion of the notion of situation. Heijmeskamp proposes that agents understand actions only in relation to situations, and develops a theoretical account that clarifies the notion of a situation. ‘Situation' is also a key topic in debating whether or not artificial entities may one day contend to the title of being agents. Jaeger et al. place the debate about organismic agency and algorithms within the discussion of situation and propose that, in contrast to algorithms, organismic agency is fundamentally agentive in that it emerges to solve the problem of what is relevant in a situation. The authors reject the idea that algorithms may lay claim to agency by arguing that discussions of agency should extend to include the context or situation as necessary in understanding what is an agent. They conclude by contending that the fundamental building blocks of cognition and consciousness are only present in natural agency and that artificial algorithmic systems do not possess genuine cognition and agency because they do not have a context in terms of any problem of relevance that they have to solve.

The theme of embodiment and its role in agency also emerges as keypoint of discussions. Exploring the social nature of agency, Achour-Benallegue et al. explore the theme of “facial icon” that usually is applied to digital face illustrations, but the authors extend it to cover a broad category of facial representations. They propose that facial icons engage social agency by triggering an embodied simulation that leads to perception of these icons as communicating not only emotions but also intentions. This opens up a new field of interdisciplinary investigation for designing such icons for purposes of social agentive engagement. Gangopadhyay and Pichler explore the topic of humans as embodied agents in a digital world that is increasingly under the influence of technologies built on non-embodied algorithms. They return to long-standing debates between 4E cognition and its critiques and propose that while it may appear that non-embodied cognition views have an explanatory advantage in the context of technologies built on non-embodied algorithms, the real contribution of these technologies to the debates is that digital technologies have great potential to uncover hitherto unexplored aspects of the mind-body continuum. Exploring these aspects has the potential to transform the debates between 4E cognition and non-embodied cognition views by revealing new ways in which digital technology can interact with and shape embodied minds.

Thus the Research Topic has engaged scholars from diverse disciplines and diverse perspectives in exploring the theme of agency in an increasingly complex world, especially in view of humanity's role and capacity to make changes for the better. We hope the Topic will encourage further interdisciplinary research that asks critical questions and provides useful answers in the face of global challenges.

